# Correlation analysis of obesity phenotypes with leptin and adiponectin

**DOI:** 10.1038/s41598-023-43550-8

**Published:** 2023-10-18

**Authors:** Guliqiekeran Aisike, Maierheba Kuerbanjiang, Dina Muheyati, Kaibinuer Zaibibuli, Mei-Xia Lv, Jia Han

**Affiliations:** Department of Nutrition and Food Hygiene, College of Public Health, Xinjiang Medical University, Urumqi, 830011 China

**Keywords:** Endocrinology, Health care

## Abstract

Obesity can be categorized as metabolically healthy obesity (MHO) and metabolically unhealthy obesity (MUO). However, individuals with MHO are characterized by the absence of metabolic syndrome (MS) and appear to have lower inflammation levels compared to MUO. This study aimed to investigate the association of obesity phenotypes with leptin (LEP) and adiponectin (ADP). According to the inclusion and exclusion criteria, we selected 178 subjects from the previous cross-sectional survey. Based on the body mass index (BMI) and diagnostic criteria of MS, we divided the individuals into three groups, including healthy control group (HC group), metabolically healthy obesity group (MHO group) and metabolically unhealthy obesity group (MUO group). The concentrations of LEP and ADP in serum were measured, and the association of these two cytokines with different obesity phenotypes were subsequently analyzed. Compared to both the HC and MHO groups, the MUO group showed significantly higher BMI, waist circumference (WC), waist-hip ratio (WHR), triglyceride (TG), total cholesterol (TC), low-density lipoprotein cholesterol (LDL-C), fasting plasma glucose (FPG), homeostasis model assessment of insulin resistance (Homa-IR) and blood pressure (*P* < 0.05). In contrast, serum high-density lipoprotein cholesterol (HDL-C) was notably lower in the MUO group (*P* < 0.05). ADP was found to have a positive correlation with systolic blood pressure (SBP) and a negative correlation with FPG in the MHO group. In the MUO group, LEP demonstrated a positive correlation with fasting insulin (FINS) and Homa-IR, while ADP showed a positive correlation with TC and SBP. Linear regression analysis further indicated that SBP (β = 0.234, *P* = 0.043), TG (β = − 0.292, *P* = 0.001) and LDL-C (β = 0.626, *P* = 0.000) were independently correlated with ADP, and BMI (β = 0.398, *P* = 0.002) was independently correlated with LEP in obese individuals. In conclusion, ADP and LEP were closely related with glucose and lipid metabolism in obese individuals, these two cytokines might play critical roles in obesity-associated metabolic disorders.

## Introduction

As of 2016, the global obesity epidemic affected over 650 million adults, including 11% of males and 15% of females^1^. Considered as a common complication of obesity, metabolic syndrome (MS) is closely associated with the development of chronic diseases^2,3^. Interestingly, not all obese individuals present with metabolic abnormalities such as hypertension, hyperlipidemia, diabetes and insulin resistance, which has been called “metabolically healthy obesity” (MHO)^4,5^. MHO is viewed as a potential transitional phase towards metabolically unhealthy obesity (MUO) which increasing the risk of negative clinical outcomes and mortality^3,6,7^.

Despite growing interest, the underling mechanisms of MHO remain unclear, and universally accepted criteria for its identification remain controversial^8^. Individuals with MHO did not display metabolic abnormalities such as hypertension, hyperlipidemia, and hyperglycemia, despite having comparable levels of total excess body fat. MS has been defined by the presence of two or more of the risk determinants as following: (1) hypertension (≥ 130/ ≥ 85 mmHg); (2) low HDL cholesterol (< 1.04 mmol/L); (3) triglycerides ≥ 1.70 mmol/L; (4) impaired fasting glucose (> 6.1 mmol/L) or diabetes; and (5) increased waist circumference (WC, male ≥ 90 cm, female ≥ 85 cm). The diagnosis of MHO requires individuals to exhibit a body mass index (BMI) high enough to qualify as obese (BMI ≥ 30 kg/m^2^) and meet zero or one of the MS criteria (excluding WC)^5,8^. However, some research suggested that the inflammatory state should also be considered when characterizing MHO. Obesity is associated with low-grade systemic inflammation, which could elevate the risk of type 2 diabetes, cardiovascular disease and other chronic diseases^9,10^. Studies showed that inflammatory markers were lower in individuals with MHO compared to those with MUO, but higher than in healthy controls^11,12^. Adipokines could contribute to the body’s inflammatory processes, and causing obesity-related complications^13^. Adipocytes, in conjunction with other inflammatory cells, could secrete proinflammatory and anti-inflammatory cytokines^14^.

Leptin (LEP), mainly secreted by adipocytes, could suppress appetite, increase energy expenditure, promote glucose utilization, improve insulin sensitivity, and exhibit a positive correlation with fat mass^15,16^. Adiponectin (ADP), another anti-inflammatory hormone secreted by adipocytes, may prevent the occurrence of cardiometabolic complications related to obesity^13,17^. ADP has physiological functions such as anti-atherosclerosis, insulin sensitization, lipid oxidation enhancement, and vasodilation^16^. Studies showed that individuals with MHO exhibited lower levels of LEP and higher levels of ADP compared to those with MUO^13,18^. However, the expression levels of inflammatory factors like ADP and LEP across different obesity phenotypes remain unclear. Therefore, this study aimed to compare the levels of LEP and ADP across different obesity phenotypes and healthy controls, and further investigated the association between these adipokines with different obesity phenotypes.

## Methods

### Study participants

**Figure 1:**
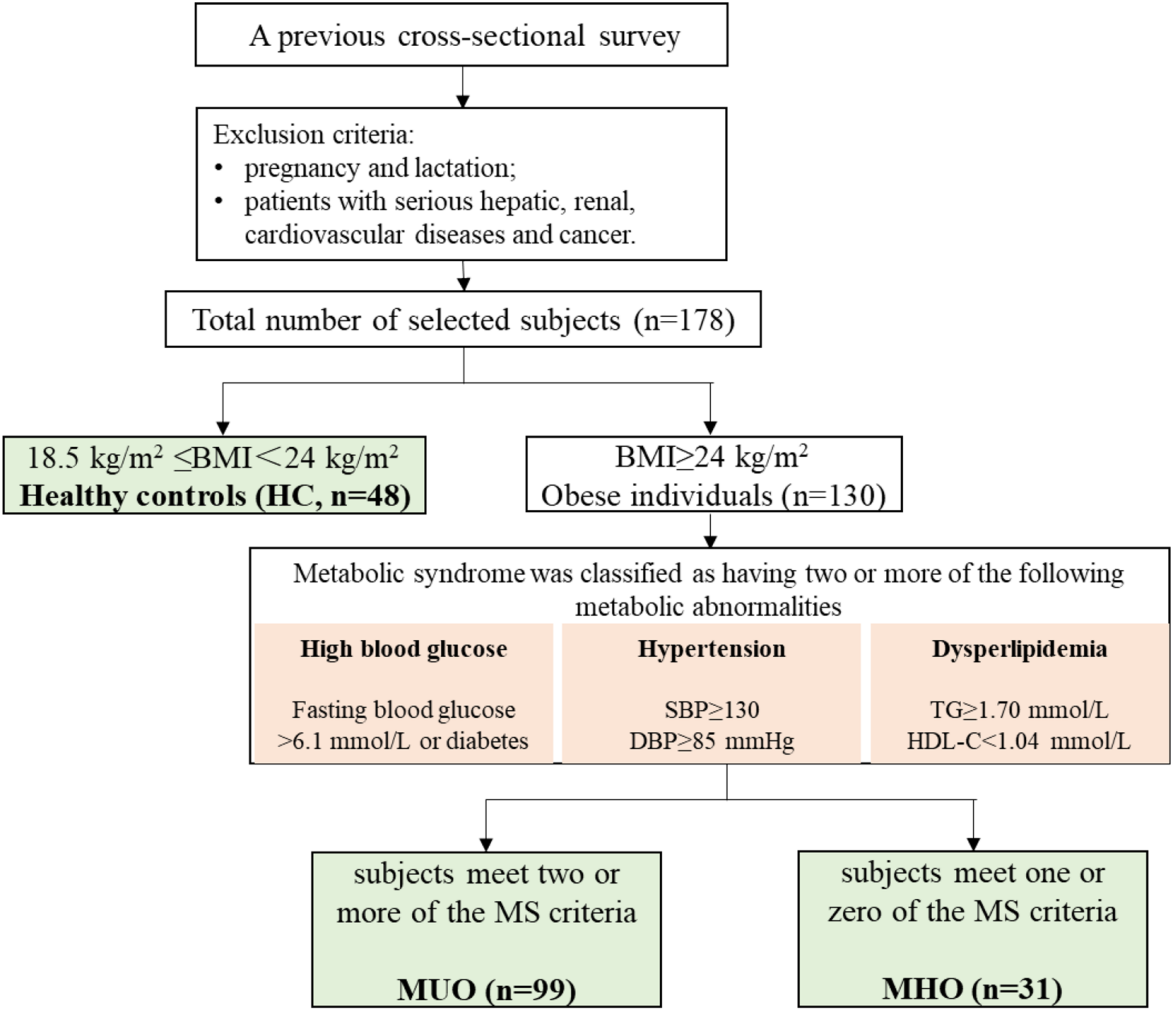
Flow chart of the study. HC, healthy controls (n = 48); MUO, metabolically unhealthy obese individuals (n = 99); MHO, metabolically healthy obese individuals (n = 31).

According to the inclusion and exclusion criteria, we selected 178 subjects from the previous cross-sectional survey which conducted in Hetian County, Xinjiang, in August 2019. The subjects were divided into three groups based on their BMI and diagnostic criteria of MS (excluding WC). The healthy control group (HC group) included 48 metabolically healthy individuals with normal-weight (18.5 kg/m^2^ ≤ BMI < 24 kg/m^2^). The metabolically unhealthy obesity group (MUO group) included 99 subjects with a BMI ≥ 24 kg/m^2^ and two or more diagnostic criteria of MS. The metabolically healthy obesity group (MHO group) consisted of 31 subjects with a BMI ≥ 24 kg/m^2^ and one or zero diagnostic criteria of MS. The exclusion criteria: pregnancy and lactation; patients with serious hepatic, renal, cardiovascular diseases and cancer (Fig. 1).

Subjects' sex and age were recorded. Anthropometric indices such as weight, height and WC, as well as blood pressure were measured, then the BMI and waist hip ratio (WHR) were calculated.

### Definition of the obesity phenotype

The parameters for BMI and WC were established according to the weight criteria for Chinese adults (2013 Edition)^19^. The diagnosis of MS was based on the guidelines of International Diabetes Federation (Table 1)^20^.

**Table 1:** Diagnostic criteria for metabolic syndrome.

Risk factor	Diagnostic level
Central obesity	Waist circumference (WC)
Male	≥ 90 cm
Female	≥ 85 cm
Triglyceride (TG)	≥ 150 mg/dl L (1.70 mmol/L)
High Density lipoprotein-cholesterol (HDL-C)	
	< 40 mg/dl L (1.04 mmol/L)
Blood pressure	≥ 130/85 mmHg
Fasting plasma glucose (FPG)	> 100 mg/dl (6.1 mmol/L)

### Determination of blood lipid and glucose concentrations

3 mL of morning fasting blood were drawn from each subject and sent for detection. The concentrations of serum triglyceride (TG), total cholesterol (TC), high-density lipoprotein cholesterol (HDL-C) and low-density lipoprotein cholesterol (LDL-C) were determined by automatic biochemistry analyzer (BS-460, Mindray, Shenzhen, China). The concentrations of fasting plasma glucose (FPG) and fasting insulin (FINS) were determined by automatic biochemistry analyzer (BS-800 M, Mindray, Shenzhen, China). The homeostasis model assessment of insulin resistance (Homa-IR) was calculated using following equation.

$$ {\text{Homa}} - {\text{IR}} = \frac{{{\text{FPG}} \times {\text{FINS}}}}{22.5} $$

### Determination of ADP and LEP concentrations

ADP and LEP were quantified using E-EL-H0004-96 T human ADP/Acrp30 (adiponectin) enzyme-linked immunosorbent assay (ELISA) kit (BLKAZ5BYNR, Elabscience, Wuhan, China) and E-EL-H0113-96 T human LEP (leptin) ELISA kit (EKF8PFBUAG, Elabscience, Wuhan, China) according to the manufacturer’s instructions.

### Statistical analysis

Data were presented as mean ± standard deviation (SD) or percentiles (P_25_, P_75_). P_25_ and P_75_ were short for the 25th and 75th percentiles, respectively. Comparison between groups was conducted by *t-*test and rank sum test. Pearson correlation analysis was used for correlation analysis, and the linear regression analysis was used for regression analysis. The data, did not show an abnormal distribution, were analyzed after logarithmic transformation. All statistical analyses were conducted using SPSS 26.0 software, with a *P*-value less than 0.05 regarded as statistically significant.

### Ethical aspects

This study has been approved by the Ethics Committee of the First Affiliated Hospital of Xinjiang Medical University (approval number: 20170214–150). All subjects were informed about the objectives of the study, guarantee of anonymity and nonuse of the data for other purposes. All participants signed informed consents. All research procedures were performed in accordance with the Declaration of Helsinki and other relevant guidelines and institutional regulations applied for studies involving human participants.

## Results

### Baseline characteristics of participants

The baseline characteristics of the subjects are listed in Table 2. A total of 178 subjects, including 73 males (41%) and 105 females (59%), with a mean age of 41.89 ± 11.42 years and a mean BMI of 27.33 ± 4.58 kg/m^2^ were investigated. Sex and age did not show significant difference among all groups. Compared to the HC group, individuals in the MHO group had significantly higher levels of BMI, WC, WHR, FINS, Homa-TR and LEP (*P* < 0.05). Compared to the MHO and HC groups, the individuals in the MUO group showed higher levels of BMI, WC, WHR, TG, TC, LDL-C, FPG, Homa-IR, systolic blood pressure (SBP), diastolic blood pressure (DBP) and lower level of HDL-C (*P* < 0.05).

**Table 2:** The baseline characteristics of the participants.

Variables	HC group (n = 48)	MHO group (n = 99)	MUO group (n = 31)	F/H ratio	*P* value
Age (years)	40.42 ± 13.14	41.43 ± 10.06	45.48 ± 12.18	5.708	0.058
Sex (n, %)				1.558	0.459
Male	19 (25.7)	39 (52.7)	16 (21.6)		
Female	29 (27.9)	60 (57.7)	15 (14.4)		
BMI (kg/m^2^)	21.59 ± 1.35	28.99 ± 3.33^a^	30.69 ± 3.37^ab^	127.31	< 0.001
WC (cm)	79.63 ± 8.14	96.35 ± 9.65^a^	104.68 ± 7.26^ab^	88.02	< 0.001
WHR	0.84 ± 0.08	0.90 ± 0.08^a^	0.96 ± 0.06^ab^	24.595	< 0.001
TG (mmol/L)	1.37 ± 0.68	1.60 ± 1.06	2.91 ± 1.65^ab^	21.180	< 0.001
TC (mmol/L)	4.26 ± 0.57	4.52 ± 1.00	4.92 ± 0.95^ab^	5.192	< 0.001
HDL-C (mmol/L)	1.55 ± 0.35	1.48 ± 0.50	1.07 ± 0.36^ab^	12.679	< 0.001
LDL-C (mmol/L)	2.41 (2.01, 2.41)	2.41 (1.56, 2.87)	2.93 (2.24, 3.66)^ab^	11.973	0.003
FPG (mmol/L)	4.80 ± 0.80	5.15 ± 0.81	5.79 ± 2.24^ab^	6.641	0.002
FINS	8.62 ± 2.39	10.07 ± 4.19^a^	11.29 ± 6.09^a^	4.034	0.019
Homa-IR	1.86 ± 0.66	2.32 ± 1.03^a^	2.90 ± 1.75^ab^	8.315	< 0.001
Blood pressure					
SBP (mmHg)	110.25 ± 16.97	113.55 ± 18.14	128.84 ± 18.12^ab^	11.34	< 0.001
DBP (mmHg)	69.52 ± 10.44	72.82 ± 10.82	82.84 ± 11.47^ab^	14.93	< 0.001
Inflammatory cytokine					
LEP	8.16 (6.48, 9.86)	8.35 (8.18,17.48)^a^	8.20 (8.15, 14.98)	16.602	< 0.001
ADP	697.40 (345.57, 794.12)	680.54 (241.56, 838.22)	710.62 (104.56, 843.59)	0.160	0.916

^a^Indicates a significant difference compared with the HC group.

^b^Indicates a significant difference compared with the MHO group.

### Correlation analysis of LEP and ADP with related metabolic indices in different obesity phenotypes

The results of the correlation analysis are listed in Table 3. In the HC group, LEP was positively correlated with TG (r = 0.332, *P* = 0.024) and DBP (r = 0.358, *P* = 0.014), and ADP was negatively correlated with TG (r = − 0.459, *P* = 0.001), TC (r = − 0.358, *P* = 0.012), SBP (r = − 0.302, *P* = 0.042), DBP (r = − 0.339, *P* = 0.021) and FINS (r = − 0.334, *P* = 0.021). In the MHO group, ADP was positively correlated with SBP (r = 0.221, *P* = 0.030) and negatively correlated with FPG (r = − 0.231, *P* = 0.023). For the MUO group, LEP was positively correlated with FINS (r = 0.572, *P* = 0.001) and Homa-IR (r = 0.457, *P* = 0.010), and ADP was positively correlated with TC (r = 0.393, *P* = 0.029), SBP (r = 0.428, *P* = 0.021).

**Table 3:** The baseline characteristics of the participants.

Index	LEP		ADP	
	r	*P*	r	*P*
TG (mmol/L)				
HC	0.332	0.024	− 0.459	0.001
MHO	0.016	0.876	− 0.177	0.082
MUO	− 0.246	0.198	− 0.222	0.246
HDL-C (mmol/L)				
HC	0.034	0.820	− 0.131	0.387
MHO	− 0.024	0.815	− 0.120	0.241
MUO	0.148	0.443	0.006	0.974
TC (mmol/L)				
HC	0.182	0.215	− 0.358	0.012
MHO	− 0.093	0.361	0.073	0.475
MUO	0.037	0.842	0.393	0.029
FPG (mmol/L)				
HC	− 0.182	0.227	0.052	0.729
MHO	− 0.140	0.170	− 0.231	0.023
MUO	− 0.019	0.923	− 0.124	0.522
SBP (mmHg)				
HC	0.190	0.205	− 0.302	0.042
MHO	0.088	0.390	0.221	0.030
MUO	0.237	0.216	0.428	0.021
DBP (mmHg)				
HC	0.358	0.014	− 0.339	0.021
MHO	0.059	0.567	0.182	0.075
MUO	0.204	0.289	− 0.006	0.975
FINS				
HC	0.197	0.180	− 0.334	0.021
MHO	0.129	0.205	− 0.060	0.554
MUO	0.572	0.001	0.240	0.193
Homa-IR				
HC	0.070	0.637	− 0.201	0.170
MHO	0.033	0.748	− 0.143	0.158
MUO	0.457	0.010	0.069	0.714

### Regression analysis of LEP and ADP levels in different obesity phenotypes

In obese individuals, SBP (β = 0.234, *P* = 0.043), TG (β = − 0.292, *P* = 0.001) and LDL-C (β = 0.626, *P* = 0.000) were independently correlated with ADP, and BMI (β = 0.398, *P* = 0.002) was independently correlated with LEP (Table 4). No significant differences were observed in other factors.

**Table 4:** Linear regression analysis of the influencing factors of LEP, ADP level in obese people.

Factor	Unstandardized coefficient		standardized coefficients	t	*P* value
	B	SE	β		
LEP					
BMI	1.574	0.486	0.398	3.241	0.002
ADP					
SBP	3.942	1.927	0.234	2.046	0.043
TG	− 70.369	21.585	− 0.292	− 3.260	0.001
LDL-C	207.743	42.990	0.626	4.832	0.000

## Discussion

In the past decade, the prevalence of obesity-related metabolic diseases has increased with economic growth and enhanced living standards in China. Obesity can be categorized as metabolically healthy obesity (MHO) and metabolically unhealthy obesity (MUO). Individuals with MHO did not display metabolic abnormalities and appeared to have lower inflammation levels compared to individuals with MUO. Several studies observed the highest values of BMI, WC, TG, Homa-IR, SBP, DBP, FINS and the lowest level of HDL-C in the MUO group compared to people with MHO and normal weight^21–23^, it is consistent with our results. Elevation levels of FINS and Homa-IR were observed both in MHO and MUO groups, indicating that the presence of insulin resistance and subsequent hyperinsulinemia. However, the MUO group had significantly higher levels of FPG and Homa-IR compared to the MHO group. Studies showed that individuals with MUO were more likely to develop type 2 diabetes compared to individuals with MHO^6,24^. Significant differences were also noted in the blood pressure between MHO and MUO groups, with research suggesting a correlation between increased BMI and increased risk for hypertension^25^.

Adipose tissue, functioning as a crucial endocrine organ, could contribute significantly to obesity-induced metabolic diseases via adipokine secretion and engagement in the immune response^26^. Disruption of the inflammatory balance may lead to a series of chronic metabolic disorders, especially obesity, type 2 diabetes, and cardiovascular disease^27^. Hotamisligil et al. highlighted the strong integration of immune response and metabolic regulation, dysfunction of which could lead to a cluster of chronic metabolic disorders^28^. Metabolic inflammation could increase the levels of inflammatory factors and maintain the body in a state of chronic low-grade inflammation^29^.

Alterations in ADP level could directly modulate lipid and glucose metabolism, further increase the synthesis of lipids, free fatty acids, and inflammatory factors. As a marker for metabolic syndrome, ADP exhibited inverse correlation with WC, visceral fat, blood pressure, blood lipids, blood glucose, and insulin levels^30–34^. Certain studies reported an elevation in serum ADP level in MHO individuals compared to MUO^18,35^. However, some researches revealed no significant difference in serum ADP levels between MHO and MUO subjects^13,21^, it is consistent with our result. We also observed that ADP was negatively correlated with FPG (r = − 0.231, *P* = 0.023) in the MHO group, as well as influencing factors like SBP, TG and LDL-C were independently correlated with ADP in obese individuals. A study also reported that serum ADP level was negatively correlated with FPG in type 2 diabetes mellitus (T2DM), and was independently associated with serum TG and LDL-C levels^36^. It indicted that a decreased in ADP was closely related to glucose metabolism and lipid metabolism.

LEP could regulate food intake and body weight, as well as play a role in proinflammatory immune responses, angiogenesis, and lipolysis. As a metabolic syndrome marker, leptin exhibited positive correlation with BMI, WC, WHR and fat mass^32,37–39^. Elevation level of LEP was observed in the MHO group, which exhibited positive correlation with FINS (r = 0.572, *P* = 0.001) and Homa-IR (r = 0.457, *P* = 0.010) in the MUO group. And BMI was independently correlated with LEP in obese subjects. Other studies also observed that LEP was positively correlated with BMI, FINS and Homa-IR^40,41^, suggesting that elevation in LEP in the MUO group is associated with glucose metabolism disorders attributable to high BMI values. LEP levels could increase with adipocyte gain and bind to the leptin receptor (LEP-R), leading to the inhibition of food intake and enhanced energy expenditure^42^. Study demonstrated that glucose transport and metabolism are key factors in the regulation of leptin expression and secretion and that the effect of insulin to increase adipocyte glucose utilization is likely to contribute to insulin-stimulated leptin secretion^43^.

## Conclusion

In this study, BMI, WC, WHR, TG, TC, LDL-C, FPG, FINS, Homa-IR and blood pressure in the MUO group were significantly higher than those in the MHO group. Results suggested that MUO is more likely to cause glucose and lipid metabolism disorders compared to MHO. Despite the observation that there were no significant differences in the levels of ADP and LEP between the individuals with MUO and MHO, these two adipokines were associated with glucose and lipid metabolism parameters in obese individuals. A decrease in ADP levels and/or an increase in LEP levels may cause metabolic disorders resultant from obesity. Therefore, it is necessary to investigate the explore association of ADP and LEP with different obesity phenotypes.

## Data Availability

The data presented in this study are available on request from the corresponding author.
